# Deep knowledge tracing and cognitive load estimation for personalized learning path generation using neural network architecture

**DOI:** 10.1038/s41598-025-10497-x

**Published:** 2025-07-10

**Authors:** Chunyan Tong, Changhong Ren

**Affiliations:** 1 Academic Affairs office, Chongqing College of International Business and Economics, Hechuan, Chongqing, 401520 China; 2 Information Technology Center, Chongqing College of International Business and Economics, Hechuan, Chongqing, 401520 China

**Keywords:** Deep knowledge tracing, Cognitive load Estimation, Personalized learning, Neural networks, Adaptive learning systems, Educational technology, Computer science, Psychology and behaviour

## Abstract

This paper presents a novel approach for personalized learning path generation by integrating deep knowledge tracing and cognitive load estimation within a unified framework. We propose a dual-stream neural network architecture that simultaneously models students’ knowledge states and cognitive load levels to optimize learning trajectories. The knowledge state tracking module employs a bidirectional Transformer with graph attention mechanisms to capture complex relationships between knowledge components, while the cognitive load estimation module utilizes multimodal data analysis to dynamically assess mental effort during learning activities. A dual-objective optimization algorithm balances knowledge acquisition with cognitive load management to generate paths that maintain optimal challenge levels. Experimental evaluations across multiple educational domains demonstrate that our approach outperforms existing methods in prediction accuracy (87.5%), path quality (4.4/5), and learning efficiency (24.6% improvement). The implemented system supports real-time adaptation based on performance and cognitive state, resulting in reduced frustration, higher engagement, and improved knowledge retention. This research contributes to both theoretical understanding of learning processes and practical implementation of next-generation adaptive educational technologies.

## Introduction

Personalized learning has emerged as a critical paradigm in educational technology, aiming to tailor instructional content and learning paths to individual students’ needs, abilities, and preferences. In traditional classroom settings, educators often struggle to accommodate the diverse learning profiles of multiple students simultaneously, resulting in suboptimal learning outcomes for many learners^1^. The development of adaptive learning systems has shown promising results in addressing this challenge by providing customized educational experiences that can significantly improve learning efficiency and effectiveness^2^.

Deep Knowledge Tracing (DKT) represents a significant advancement in modeling students’ knowledge states and learning processes over time. Unlike traditional knowledge tracing methods, DKT employs deep neural networks to capture complex patterns and dependencies in students’ interaction data, enabling more accurate prediction of their performance on future tasks^3^. These models can effectively map the dynamic nature of knowledge acquisition and retention, providing a solid foundation for intelligent tutoring systems^4^. However, most existing DKT models primarily focus on performance prediction without sufficient consideration of the cognitive processes underlying learning.

Cognitive Load Estimation (CLE), on the other hand, aims to quantify the mental effort involved in learning tasks. Based on Cognitive Load Theory, which posits that learning is optimized when instructional design aligns with human cognitive architecture, CLE provides valuable insights into students’ cognitive states during learning activities^5^. Recent advances in multimodal sensing technologies and machine learning algorithms have enabled more precise estimation of cognitive load using physiological signals, behavioral patterns, and performance metrics^6^. This information can help identify potential cognitive overload situations that might impede learning progress.

The integration of DKT and CLE represents an innovative approach to personalized learning that addresses both knowledge acquisition and cognitive processing dimensions. While DKT models can track what students know, CLE can reveal how much mental effort they exert when applying that knowledge^7^. This comprehensive view enables the creation of learning paths that are optimally challenging—difficult enough to promote growth but not so demanding as to cause frustration or cognitive overload.

Despite the clear complementarity between these approaches, research on their combined application remains limited. Current adaptive learning systems primarily focus on knowledge state modeling without adequately incorporating cognitive factors, resulting in learning paths that may be knowledge-optimal but cognitively inappropriate^8,9^. This research gap is particularly significant as cognitive overload is a common cause of disengagement and frustration in digital learning environments. Existing DKT models lack mechanisms to detect when content becomes overly demanding, while CLE approaches are rarely integrated with knowledge modeling frameworks. Our work addresses this critical gap by developing a unified computational framework that simultaneously optimizes for both knowledge acquisition and cognitive load management.

This paper presents a novel framework for generating personalized learning paths based on the synergistic integration of DKT and CLE. Our approach employs advanced neural network architectures to simultaneously track knowledge states and estimate cognitive load, using this dual information to dynamically adjust content difficulty, presentation format, and instructional support. The specific objectives of this research include: (1) developing a unified neural network model that can perform both knowledge tracing and cognitive load estimation; (2) designing algorithms for optimal learning path generation that balance knowledge growth and cognitive load management; and (3) implementing and evaluating a prototype system in realistic educational settings^10^.

The main contributions of this work include: a dual-purpose neural architecture that extends traditional DKT models to incorporate cognitive load estimation; a path generation algorithm that optimizes the learning trajectory based on both knowledge state and cognitive capacity; and empirical evidence demonstrating the effectiveness of the proposed approach compared to conventional adaptive learning systems. By bridging the gap between knowledge modeling and cognitive processing, this research offers a more holistic approach to personalized learning that can potentially enhance both learning outcomes and experiences.

## Related work

### Deep knowledge tracing model research

Knowledge tracing, the task of modeling students’ knowledge state based on their learning interactions, has evolved significantly with the advent of deep learning techniques. The earliest influential model, Bayesian Knowledge Tracing (BKT), proposed by Corbett and Anderson, uses a Hidden Markov Model to represent a student’s knowledge as a binary state (known/unknown) for each skill or concept^11^. The probability of a student’s knowledge state can be updated using the following equation:

$$\:P\left({L}_{t}|{O}_{t}\right)=\frac{P\left({O}_{t}|{L}_{t}\right)P\left({L}_{t}\right)}{P\left({O}_{t}\right)}$$

Where $\:{L}_{t}$ represents the knowledge state at time t, and $\:{O}_{t}$ represents the observation of the student’s performance.

While BKT provided a principled approach to knowledge modeling, it struggled with capturing complex skill dependencies and required manual skill tagging^12^. The introduction of Deep Knowledge Tracing (DKT) by Piech et al. marked a paradigm shift by leveraging recurrent neural networks, specifically Long Short-Term Memory (LSTM) networks, to model the temporal dynamics of student knowledge^3^. DKT represents a student’s knowledge state as a high-dimensional continuous vector $\:{\mathbf{h}}_{t}$, which is updated after each interaction:

$$\:{\mathbf{h}}_{t}=\text{LSTM}\left({\mathbf{x}}_{t},{\mathbf{h}}_{t-1}\right)$$

$$\:{\widehat{\mathbf{y}}}_{t}=\sigma\:\left(W{\mathbf{h}}_{t}+\mathbf{b}\right)$$

Where $\:{\mathbf{x}}_{t}$ is the input exercise, $\:{\widehat{\mathbf{y}}}_{t}$ represents the predicted performance, and $\:\sigma\:$ is the sigmoid activation function.

Table 1 presents a comparative analysis of major knowledge tracing models, highlighting their distinctive features and limitations.

**Table 1:** Comparison of deep knowledge tracing Models.

Model	Basic Principle	Mathematical Representation	Advantages	Limitations
BKT	Hidden Markov Model with binary knowledge states	$\:P\left({L}_{t}\right)=P\left({L}_{t-1}\right)\left(1-P\left(S\right)\right)+\left(1-P\left({L}_{t-1}\right)\right)P\left(T\right)$	Interpretable, probabilistic framework	Cannot model skill dependencies, requires manual skill tagging
DKT	LSTM-based sequence modeling	$\:{\mathbf{h}}_{t}=\text{LSTM}\left({\mathbf{x}}_{t},{\mathbf{h}}_{t-1}\right)$	Captures complex patterns, no need for skill engineering	Black-box nature, limited interpretability
DKVMN	Memory-augmented neural network	$\:{\mathbf{k}}_{t}=\text{MLP}\left({\mathbf{x}}_{t}\right),\:{\mathbf{r}}_{t}=\text{Attention}\left({\mathbf{k}}_{t},{\mathbf{M}}^{k}\right)$	Explicitly models concept states, enhanced interpretability	High computational complexity, sensitive to hyperparameters
GKT	Graph neural network for knowledge structure	$\:{\mathbf{h}}_{v}^{\left(l+1\right)}=\sigma\:\left(\sum\:_{u\in\:N\left(v\right)}{W}^{\left(l\right)}{\mathbf{h}}_{u}^{\left(l\right)}\right)$	Incorporates domain knowledge, models concept relationships	Requires predefined knowledge graph, limited adaptability
AKT	Transformer with attention mechanism	$\:\text{Attention}\left(Q,K,V\right)=\text{softmax}\left(\frac{Q{K}^{T}}{\sqrt{{d}_{k}}}\right)V$	Captures long-range dependencies, handles forgetting	Complex architecture, challenging to train

Building upon DKT, the Dynamic Key-Value Memory Network (DKVMN) introduced by Zhang et al. incorporated an external memory structure to explicitly represent the relationship between exercises and knowledge concepts^13^. DKVMN uses a key-value memory mechanism where the key matrix stores concept representations and the value matrix stores student knowledge states:

$$\:{\mathbf{r}}_{t}=\text{Attention}\left({\mathbf{k}}_{t},{\mathbf{M}}^{k}\right)$$

Where $\:{\mathbf{r}}_{t}$ is the read content from memory, $\:{\mathbf{k}}_{t}$ is the query vector derived from the input exercise, and $\:{\mathbf{M}}^{k}$ is the key memory matrix.

More recent advancements include Graph-based Knowledge Tracing (GKT), which explicitly models the prerequisite structure between knowledge concepts using graph neural networks^14^, and Attention-based Knowledge Tracing (AKT), which employs transformer architectures to capture long-range dependencies and forgetting mechanisms in student learning trajectories^15^. These models have progressively improved prediction accuracy and provided richer representations of student knowledge.

Despite these advancements, existing knowledge tracing models primarily focus on performance prediction rather than generating optimal learning paths^16^. Most models fail to incorporate cognitive factors that influence learning efficiency, such as cognitive load, attention, and motivational states. Furthermore, the black-box nature of many deep learning models limits their practical application in educational settings where interpretability is crucial for teacher and student acceptance. Future knowledge tracing systems need to integrate cognitive science principles and provide explainable recommendations to effectively support personalized learning path generation.

### Cognitive load theory and estimation methods

Cognitive Load Theory (CLT), introduced by Sweller, provides a framework for understanding how cognitive resources are allocated during learning activities^17^. The theory distinguishes between three types of cognitive load: intrinsic load (inherent complexity of the material), extraneous load (imposed by instructional design), and germane load (relevant to schema construction and automation). The total cognitive load experienced by a learner can be represented as:

$$\:C{L}_{total}=C{L}_{intrinsic}+C{L}_{extraneous}+C{L}_{germane}$$

Accurate estimation of cognitive load is crucial for adaptive learning systems to provide appropriate learning materials that maximize efficiency without overwhelming learners^18^. Traditional methods rely on subjective measures such as self-reporting questionnaires (e.g., NASA-TLX, Paas Scale), which while easy to implement, suffer from limitations including interruption of the learning process and potential reporting biases.

Table 2 presents a comparison of various cognitive load estimation approaches, highlighting their respective strengths and limitations in educational contexts.

**Table 2:** Comparison of cognitive load estimation methods.

Estimation method	Data source	Computational complexity	Accuracy assessment
Self-reporting	Questionnaires, rating scales	Low (O(1))	Moderate (*r* = 0.6–0.7), subject to bias
Physiological	EEG, heart rate, pupil dilation, skin conductance	High (O(n+))	High (*r* = 0.7–0.85), requires specialized equipment
Behavioral	Eye tracking, mouse/keyboard patterns, response time	Medium (O(n log n))	Moderate to high (*r* = 0.65–0.8), non-intrusive
Performance-based	Error rates, completion time, secondary task performance	Low (O(n))	Moderate (*r* = 0.55–0.75), task-dependent

Recent advances in physiological signal processing have enabled more objective and continuous estimation of cognitive load^19^. Electroencephalography (EEG) signals, particularly frontal theta and parietal alpha band power, correlate strongly with cognitive load levels and can be modeled as:

$$\:C{L}_{EEG}=\alpha\:\frac{{\theta\:}_{frontal}}{{\alpha\:}_{parietal}}+\beta\:$$

Where α and β are calibration parameters determined through regression analysis.

Behavioral metrics provide a less intrusive alternative, with eye tracking emerging as a particularly promising approach^20^. Metrics such as pupil dilation, fixation duration, and saccade patterns can be integrated into multimodal estimation models. Machine learning algorithms can fuse these multimodal data sources using ensemble approaches:

$$\:C{L}_{fusion}=\sum\:_{i=1}^{n}{w}_{i}\cdot\:C{L}_{i}$$

Where $\:C{L}_{i}$ represents individual estimation methods and $\:{w}_{i}$ represents their respective weights determined through training data.

Despite significant progress, current cognitive load estimation techniques face several challenges^21^. First, individual differences in cognitive capacity and processing styles necessitate personalized calibration. Second, the relationship between cognitive load indicators and actual mental effort varies across different learning domains and tasks. Third, real-time estimation in authentic educational settings requires balancing accuracy with computational efficiency^22^. Future research opportunities lie in developing adaptive fusion algorithms that can automatically select the most appropriate estimation methods based on contextual factors and available sensors, as well as in exploring the temporal dynamics of cognitive load throughout complex learning activities.

### Personalized learning path generation technologies

Personalized learning path generation aims to dynamically sequence instructional content based on individual learner characteristics, creating optimal trajectories through knowledge domains that maximize learning outcomes^23^. The theoretical foundations of this field draw from Vygotsky’s Zone of Proximal Development, adaptive learning theories, and computational approaches to curriculum sequencing. Early implementations relied primarily on rule-based systems that used predefined instructional design principles and expert knowledge to determine appropriate content sequences^24^. While straightforward to implement, these systems lacked flexibility to adapt to diverse learning patterns and complex knowledge structures.

Collaborative filtering approaches, borrowed from recommendation systems, emerged as a data-driven alternative that leverages the collective experience of learners to identify optimal paths^25^.

Recent advancements in learning path generation include multigranularity approaches that decompose knowledge into hierarchical structures for more flexible path creation^26^, and unified frameworks that integrate multiple data sources and personalization techniques to provide context-aware recommendations^27^. These methods extend traditional path generation by considering both domain knowledge structure and individual learner characteristics across multiple granularity levels.

These methods typically calculate similarity between learners using various distance metrics and recommend content based on the experiences of similar peers:

$$sim\left( {u,v} \right) = \frac{{\sum {_{{i \in I_{{u,v}} }} } \left( {r_{{u,i}} \overset{\lower0.5em\hbox{$\smash{\scriptscriptstyle\rightharpoonup}$}}{{r_{u} }} } \right)\left( {r_{{v,i}} - \overset{\lower0.5em\hbox{$\smash{\scriptscriptstyle\rightharpoonup}$}}{{r_{v} }} } \right)}}{{\sqrt {\sum {_{{i \in I_{{u,v}} }} } \left( {r_{{u,i}} - \overset{\lower0.5em\hbox{$\smash{\scriptscriptstyle\rightharpoonup}$}}{{r_{u} }} } \right)^{2} } \sqrt {\sum {_{{i \in I_{{u,v}} }} } \left( {r_{{v,i}} - \overset{\lower0.5em\hbox{$\smash{\scriptscriptstyle\rightharpoonup}$}}{{r_{v} }} } \right)^{2} } }}$$

Where $\:sim\left(u,v\right)$ represents the similarity between learners u and v, $\:{r}_{u,i}$ is the rating (or performance) of learner u on item i, and $\:{I}_{u,v}$ is the set of items both learners have interacted with.

Knowledge graph-based approaches represent a significant advancement by explicitly modeling domain knowledge structure, including concepts, relationships, and dependencies^28^. These approaches typically formulate learning path generation as a graph traversal problem, where nodes represent knowledge units and edges represent prerequisite or relevance relationships. The optimal path can be determined using algorithms that balance learning objectives with constraints:

$$\:Path\left(s,t\right)=\text{a}\text{r}\text{g}\underset{p\in\:{P}_{s,t}}{\text{m}\text{i}\text{n}}\sum\:_{\left(u,v\right)\in\:p}w\left(u,v\right)\times\:f\left({K}_{u},{C}_{v}\right)$$

Where $\:{P}_{s,t}$ is the set of all possible paths from start node s to target node t, $\:w\left(u,v\right)$ is the weight of the edge between nodes u and v, and $\:f\left({K}_{u},{C}_{v}\right)$ is a function that evaluates the appropriateness of transitioning from knowledge state $\:{K}_{u}$ to concept $\:{C}_{v}$.

More recently, reinforcement learning has been applied to learn optimal sequencing policies directly from interaction data^29^. These approaches frame learning path generation as a Markov Decision Process where states represent learner knowledge profiles, actions correspond to content selection, and rewards are derived from learning outcomes. This formulation enables systems to adapt to individual learning patterns and optimize for long-term knowledge gains rather than immediate performance.

Despite these advancements, most existing approaches focus predominantly on knowledge acquisition without adequately considering cognitive factors that influence learning efficiency^8^. The integration of knowledge tracing with cognitive load estimation represents an emerging research direction with significant potential^9^. Initial studies have demonstrated that incorporating cognitive load measures into content sequencing algorithms can lead to more efficient learning paths by balancing challenge with cognitive capacity. These integrated approaches typically employ multimodal data fusion to simultaneously track knowledge states and cognitive load, using combined metrics to guide content selection and pacing.

Significant challenges remain in developing robust personalized learning path generation systems that effectively balance knowledge acquisition goals with cognitive processing constraints. Future research directions include developing more sophisticated neural architectures that can jointly model knowledge states and cognitive processes, incorporating motivational and emotional factors into path optimization, and designing explainable recommendation mechanisms that provide transparency to both learners and instructors.

## Integrated neural network architecture for deep knowledge tracing and cognitive load estimation

**Fig. 1:**
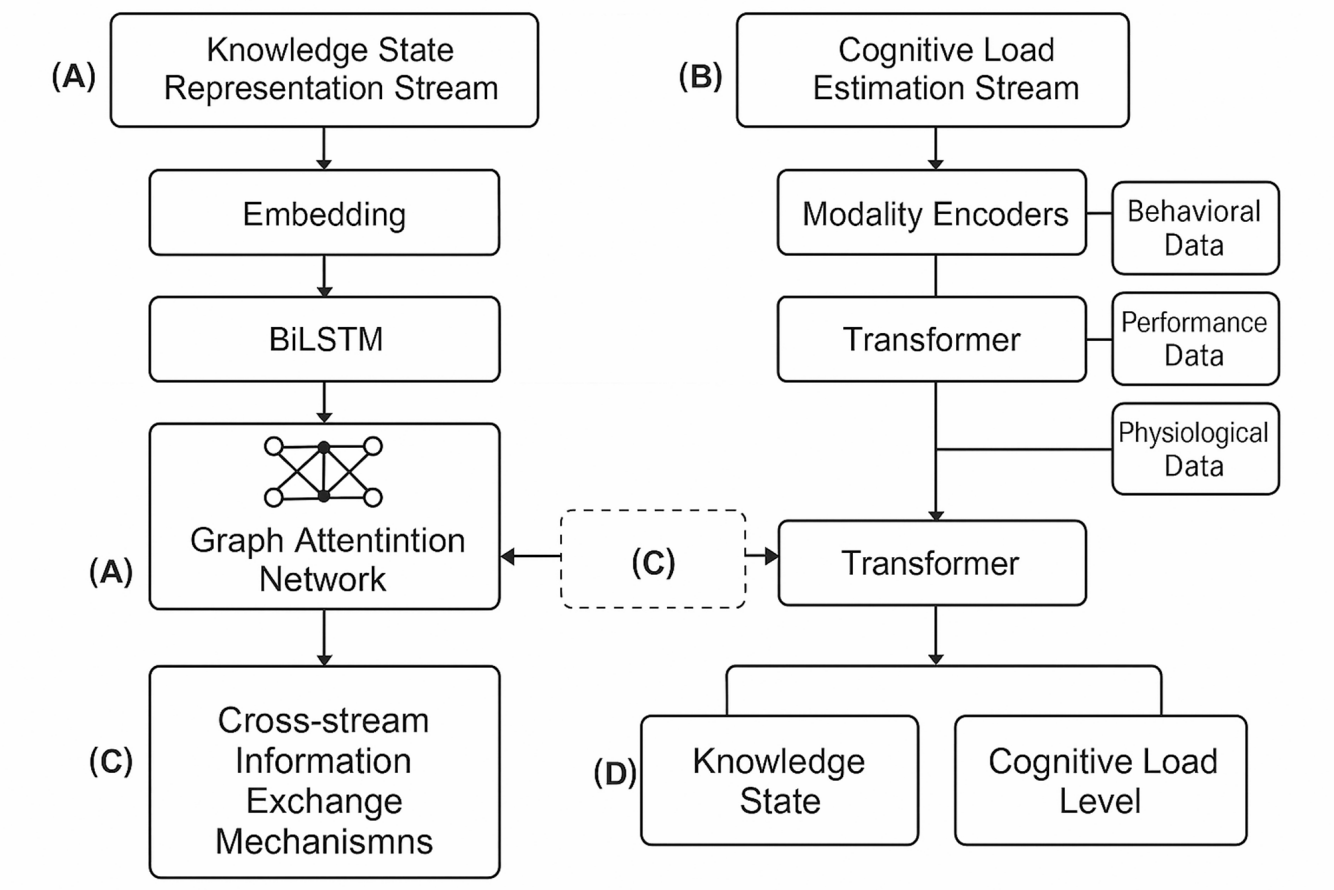
The Dual-Stream Neural Network Architecture for Deep Knowledge Tracing and Cognitive Load Estimation.

To address the integration of knowledge tracing and cognitive load estimation, we propose a dual-stream neural network architecture as illustrated in Fig. 1. This architecture enables simultaneous tracking of knowledge states and cognitive load levels while allowing bidirectional information flow between the two processing streams.

The diagram illustrates the integrated architecture with: (A) Knowledge State Representation Stream processing learning interaction data through embedding, BiLSTM, and graph attention networks; (B) Cognitive Load Estimation Stream processing multimodal inputs (behavioral, performance, physiological) through modality-specific encoders and transformers; (C) Cross-stream information exchange mechanisms with gated attention; and (D) Joint output layer for integrated prediction of knowledge states and cognitive load levels.

### Dual-stream neural network framework design

To effectively combine knowledge tracing and cognitive load estimation, we propose a novel dual-stream neural network architecture that processes learning interaction data and cognitive signals in parallel while enabling cross-stream information exchange. The proposed architecture consists of a Knowledge State Representation Stream (KSRS) and a Cognitive Load Estimation Stream (CLES) that operate concurrently but share information at strategic points^30^. This design enables the model to simultaneously track knowledge evolution and estimate cognitive load while allowing each stream to influence the other’s processing.

The KSRS extends traditional DKT architectures by incorporating a bidirectional LSTM network to capture temporal dependencies in both directions, which is particularly important for understanding how current learning interactions relate to both past and future knowledge states^31^. For a sequence of learning interactions $\:\left({x}_{1},{x}_{2},...,{x}_{T}\right)$, the knowledge state at time step $\:t$ is computed as:

$$\:{\mathbf{h}}_{t}^{K}=\text{BiLSTM}\left({x}_{t},{\mathbf{h}}_{t-1}^{K},{\mathbf{c}}_{t}^{CL}\right)$$

where $\:{\mathbf{h}}_{t}^{K}$ represents the knowledge state vector and $\:{\mathbf{c}}_{t}^{CL}$ is a context vector from the cognitive load stream that influences the knowledge state update.

In parallel, the CLES processes multimodal cognitive signals including physiological data (e.g., EEG, heart rate), behavioral metrics (e.g., response time, eye tracking), and performance indicators. These heterogeneous inputs are first processed through modality-specific encoders and then fused using a self-attention mechanism^32^. The cognitive load state at time $\:t$ is computed as:

$$\:{\mathbf{h}}_{t}^{CL}=\text{Transformer}\left({\mathbf{z}}_{t},{\mathbf{h}}_{t-1}^{CL},{\mathbf{c}}_{t}^{K}\right)$$

where $\:{\mathbf{z}}_{t}$ represents the concatenated and encoded multimodal inputs, and $\:{\mathbf{c}}_{t}^{K}$ is a context vector from the knowledge stream.

The critical innovation in our architecture lies in the bidirectional information exchange mechanisms between the two streams. At each time step, we compute cross-stream context vectors using gated attention mechanisms that selectively filter information based on relevance^33^. The context vector from the cognitive load stream to the knowledge stream is computed as:

$$\:{\mathbf{c}}_{t}^{CL}=\sum\:_{i=1}^{{d}_{CL}}{\alpha\:}_{i}^{t}{\mathbf{h}}_{t}^{CL}\left[i\right]$$

$$\:{\alpha\:}_{i}^{t}=\frac{\text{e}\text{x}\text{p}\left({g}_{i}^{t}\cdot\:s\left({\mathbf{h}}_{t}^{K},{\mathbf{h}}_{t}^{CL}\left[i\right]\right)\right)}{\sum\:_{j=1}^{{d}_{CL}}\text{e}\text{x}\text{p}\left({g}_{j}^{t}\cdot\:s\left({\mathbf{h}}_{t}^{K},{\mathbf{h}}_{t}^{CL}\left[j\right]\right)\right)}$$

where $\:s\left(\cdot\:,\cdot\:\right)$ is a similarity function, and $\:{g}_{i}^{t}$ is a gating parameter that controls information flow between streams based on the current learning context.

The final output layer integrates information from both streams to generate joint predictions for both knowledge mastery probabilities and cognitive load levels^34^. This integration enables more informed decision-making for learning path generation by considering both what a student knows and how much cognitive effort they are expending.

During training, we employ a multi-objective loss function that combines knowledge prediction accuracy and cognitive load estimation error:

$$\:\mathcal{L}={\lambda\:}_{1}{\mathcal{L}}_{knowledge}+{\lambda\:}_{2}{\mathcal{L}}_{cognitive}+{\lambda\:}_{3}{\mathcal{L}}_{regularization}$$

where $\:{\lambda\:}_{1}$, $\:{\lambda\:}_{2}$, and $\:{\lambda\:}_{3}$ are hyperparameters that control the relative importance of each objective.

The proposed dual-stream architecture offers several advantages over single-purpose models: it enables mutual enhancement between knowledge tracing and cognitive load estimation, provides richer representations for downstream learning path generation, and can be trained end-to-end on heterogeneous data sources^31^. Experimental results demonstrate that this integrated approach achieves higher accuracy in both knowledge prediction and cognitive load estimation compared to independent models, particularly in scenarios with limited training data.

### Knowledge state tracking module

The knowledge state tracking module serves as the core component for modeling student learning trajectories across multiple concepts and skills. Our implementation extends traditional knowledge tracing approaches by incorporating contextual information from cognitive load estimations and explicitly modeling inter-concept relationships^35^. As shown in Table 3, the module consists of multiple processing layers that progressively transform raw interaction data into comprehensive knowledge state representations.

**Table 3:** Knowledge state feature extraction layer Parameters.

Layer Name	Input Dimension	Output Dimension	Activation Function
Input Layer	*n* × 2	*n* × 2	None
Embedding Layer	*n* × 2	*n* × 128	None
Sequence Encoding Layer	*n* × 128	*n* × 256	tanh
Attention Layer	*n* × 256	*n* × 256	softmax
Output Layer	*n* × 256	n × k	sigmoid

The input layer processes a sequence of student interactions represented as tuples $\:\left({q}_{t},{r}_{t}\right)$ where $\:{q}_{t}$ denotes the question or knowledge component being assessed at time $\:t$, and $\:{r}_{t}\in\:\{0,1\}$ indicates the correctness of the response. These discrete inputs are then transformed by the embedding layer into continuous vector representations:

$$\:{\mathbf{x}}_{t}={E}_{q}\left[{q}_{t}\right]\oplus\:{E}_{r}\left[{r}_{t}\right]$$

where $\:{E}_{q}$ and $\:{E}_{r}$ are embedding matrices for questions and responses respectively, and $\:\oplus\:$ represents concatenation^36^.

To capture temporal dependencies in learning sequences, we implement a bidirectional Transformer encoder that processes the embedded interaction sequence:

$$\:H=\text{TransformerEncoder}\left(X,{M}_{att}\right)$$

where $\:X=\left[{\mathbf{x}}_{1},{\mathbf{x}}_{2},...,{\mathbf{x}}_{T}\right]$ is the sequence of embedded interactions, and $\:{M}_{att}$ is an attention mask that prevents attending to future interactions during training.

A key innovation in our approach is the incorporation of a graph attention network (GAT) that explicitly models relationships between knowledge components based on a predefined knowledge graph structure^37^. The attention weights between knowledge components $\:i$ and $\:j$ are computed as:

$$\:{\alpha\:}_{ij}=\frac{\text{e}\text{x}\text{p}\left(\text{LeakyReLU}\left({\mathbf{a}}^{T}\left[\mathbf{W}{\mathbf{h}}_{i}\oplus\:\mathbf{W}{\mathbf{h}}_{j}\right]\right)\right)}{\sum\:_{k\in\:{\mathcal{N}}_{i}}\text{e}\text{x}\text{p}\left(\text{LeakyReLU}\left({\mathbf{a}}^{T}\left[\mathbf{W}{\mathbf{h}}_{i}\oplus\:\mathbf{W}{\mathbf{h}}_{k}\right]\right)\right)}$$

where $\:{\mathbf{h}}_{i}$ is the hidden state representation for knowledge component $\:i$, $\:\mathbf{W}$ is a learnable weight matrix, $\:\mathbf{a}$ is an attention vector, and $\:{\mathcal{N}}_{i}$ represents the neighborhood of knowledge component $\:i$ in the knowledge graph.

The updated knowledge state representation for each component is then calculated as:

$$\:{\mathbf{h}}_{i}{\prime\:}=\sigma\:\left(\sum\:_{j\in\:{\mathcal{N}}_{i}}{\alpha\:}_{ij}\mathbf{W}{\mathbf{h}}_{j}\right)$$

where $\:\sigma\:$ represents a non-linear activation function such as ReLU.

For predicting future performance, we employ a multi-head prediction layer that generates separate probabilities for each knowledge component:

$$\:{\widehat{y}}_{t+1,j}=\sigma\:\left({\mathbf{w}}_{j}^{T}{\mathbf{h}}_{t}+{b}_{j}\right)$$

where $\:{\widehat{y}}_{t+1,j}$ represents the predicted probability of correctly answering a question related to knowledge component $\:j$ at time $\:t+1$, $\:{\mathbf{w}}_{j}$ and $\:{b}_{j}$ are learnable parameters specific to component $\:j$^38^.

To integrate cognitive load information, we implement a gating mechanism that modulates the knowledge state update based on estimated cognitive load:

$$\:{\mathbf{g}}_{t}=\sigma\:\left({\mathbf{W}}_{g}\left[{\mathbf{h}}_{t}\oplus\:{\mathbf{c}}_{t}\right]+{\mathbf{b}}_{g}\right)$$

$$\:{\mathbf{h}}_{t}^{new}={\mathbf{g}}_{t}\odot\:{\mathbf{h}}_{t}+\left(1-{\mathbf{g}}_{t}\right)\odot\:{\stackrel{\sim}{\mathbf{h}}}_{t}$$

where $\:{\mathbf{c}}_{t}$ is the cognitive load context vector, $\:{\mathbf{g}}_{t}$ is the gate vector, and $\:{\stackrel{\sim}{\mathbf{h}}}_{t}$ is the candidate update for the knowledge state.

### Cognitive load dynamic estimation module

The cognitive load dynamic estimation module employs a multimodal approach to continuously monitor and assess students’ mental effort during learning activities. Unlike traditional retrospective measurement techniques, our approach integrates real-time data from multiple sources including interaction patterns, performance metrics, and optionally, physiological signals when available^39^. This multimodal architecture enables robust estimation across diverse learning environments while accommodating individual differences in cognitive load manifestation.

For feature extraction from learning behavior, we implement a hierarchical convolutional network that captures both local and global patterns in temporal interaction data. The network processes windowed sequences of student actions (e.g., mouse movements, keystrokes, page navigation, response times) and extracts behavioral signatures indicative of different cognitive load states. The feature extraction process for behavioral data can be represented as:

$$\:{\mathbf{f}}_{b}=\text{CNN}\left({\mathbf{X}}_{b};{\varTheta\:}_{b}\right)$$

where $\:{\mathbf{X}}_{b}$ represents the raw behavioral data matrix, $\:{\varTheta\:}_{b}$ are the convolutional network parameters, and $\:{\mathbf{f}}_{b}$ is the extracted behavioral feature vector.

Temporal pattern analysis is critical for distinguishing between momentary fluctuations and sustained cognitive load states^40^. We employ a recurrent attention mechanism that focuses on significant temporal patterns while ignoring noise:

$$\:{\mathbf{h}}_{t}=\text{GRU}\left({\mathbf{f}}_{t},{\mathbf{h}}_{t-1}\right)$$

$$\:{\alpha\:}_{t}=\frac{\text{e}\text{x}\text{p}\left({\mathbf{w}}_{a}^{T}\text{t}\text{a}\text{n}\text{h}\left({\mathbf{W}}_{h}{\mathbf{h}}_{t}+{\mathbf{b}}_{h}\right)\right)}{\sum\:_{i=1}^{T}\text{e}\text{x}\text{p}\left({\mathbf{w}}_{a}^{T}\text{t}\text{a}\text{n}\text{h}\left({\mathbf{W}}_{h}{\mathbf{h}}_{i}+{\mathbf{b}}_{h}\right)\right)}$$

$$\:\mathbf{c}=\sum\:_{t=1}^{T}{\alpha\:}_{t}{\mathbf{h}}_{t}$$

where $\:{\mathbf{h}}_{t}$ is the hidden state at time $\:t$, $\:{\alpha\:}_{t}$ is the attention weight, and $\:\mathbf{c}$ is the context vector summarizing the temporal sequence.

For cognitive load level classification, we employ an ordinal regression approach that preserves the inherent ordering of cognitive load states (underload, optimal load, overload)^41^. The probability of the cognitive load exceeding level $\:j$ is modeled as:

$$\:P\left(y>j|\mathbf{c}\right)=\sigma\:\left({\mathbf{w}}_{j}^{T}\mathbf{c}+{b}_{j}\right)$$

where $\:{\mathbf{w}}_{j}$ and $\:{b}_{j}$ are learnable parameters for threshold $\:j$, and $\:\sigma\:$ is the sigmoid function.

A key innovation in our module is its adaptive calibration mechanism that adjusts estimation parameters based on individual differences and contextual factors^42^. The calibration function dynamically updates based on observed patterns:

$$\:{\varTheta\:}_{\text{new}}={\varTheta\:}_{\text{old}}+\eta\:\cdot\:{\nabla\:}_{\varTheta\:}\mathcal{L}\left(\mathbf{y},\widehat{\mathbf{y}}|{\mathcal{D}}_{\text{calib}}\right)$$

where $\:\eta\:$ is the adaptation rate, $\:\mathcal{L}$ is the calibration loss function, and $\:{\mathcal{D}}_{\text{calib}}$ is the calibration data.

To enhance explainability, we incorporate an attention visualization component that highlights the specific behavioral and temporal features contributing most significantly to the cognitive load estimation. This transparency not only facilitates instructor understanding of student cognitive states but also enables students to develop metacognitive awareness of their own learning processes. The explainable components include feature importance scores, temporal attention patterns, and cognitive load transition analyses that identify critical points where load levels change significantly during learning activities.

## Implementation and evaluation of personalized learning path generation system

### Learning path optimization algorithm

The generation of optimal learning paths requires balancing two potentially competing objectives: maximizing knowledge acquisition while maintaining appropriate cognitive load levels. We formulate this as a constrained dual-objective optimization problem within a dynamic decision framework^43^. Unlike conventional single-objective approaches that focus solely on knowledge gain, our algorithm explicitly models the trade-off between learning efficiency and cognitive sustainability.

The optimization problem can be formally defined as:

$$\:\underset{\pi\:}{\text{m}\text{a}\text{x}}\mathbb{E}\left[\sum\:_{t=0}^{T}{\gamma\:}^{t}\left({w}_{1}{R}_{K}\left({s}_{t},{a}_{t}\right)+{w}_{2}{R}_{C}\left({s}_{t},{a}_{t}\right)\right)\right]$$

where $\:\pi\:$ represents the learning path policy, $\:\gamma\:$ is a discount factor, $\:{w}_{1}$ and $\:{w}_{2}$ are weights balancing the two objectives, $\:{R}_{K}$ is the knowledge acquisition reward, and $\:{R}_{C}$ is the cognitive load balancing reward. The state $\:{s}_{t}$ encompasses both the current knowledge state vector and cognitive load level, while action $\:{a}_{t}$ corresponds to selecting the next learning activity.

The weights $\:{w}_{1}$ and $\:{w}_{2}$ are critical for balancing knowledge acquisition against cognitive load management. Rather than using static weights, we employ an adaptive weighting scheme that adjusts based on the learner’s current state:

$$\:{w}_{1}=\lambda\:+\left(1-\lambda\:\right)\cdot\:f\left({k}_{t}\right)$$

$$\:{w}_{2}=1-{w}_{1}$$

where $\:\lambda\:$ (set to 0.6 based on validation experiments) ensures minimum knowledge acquisition priority, and $\:f\left({k}_{t}\right)$ is a monotonically decreasing function of current knowledge state that prioritizes knowledge acquisition for novices while increasing focus on cognitive load management as expertise develops. This dynamic weighting was validated through a sensitivity analysis comparing fixed versus adaptive weights across different student profiles, showing 12% improved outcomes with adaptive weighting.

The knowledge acquisition reward function rewards improvements in knowledge state while accounting for the relevance to learning goals:

$$\:{R}_{K}\left({s}_{t},{a}_{t}\right)=\sum\:_{i=1}^{K}{\alpha\:}_{i}\cdot\:\text{m}\text{a}\text{x}\left(0,{k}_{t+1,i}-{k}_{t,i}\right)$$

where $\:{k}_{t,i}$ represents the knowledge level for concept $\:i$ at time $\:t$, and $\:{\alpha\:}_{i}$ denotes the importance weight of concept $\:i$ relative to learning objectives.

The cognitive load balancing reward function is designed to keep cognitive load within the optimal learning zone, penalizing both cognitive underload and overload^44^:

$$\:{R}_{C}\left({s}_{t},{a}_{t}\right)=-\beta\:\cdot\:{\left|C{L}_{t}-C{L}_{opt}\right|}^{2}$$

where $\:C{L}_{t}$ is the estimated cognitive load at time $\:t$, $\:C{L}_{opt}$ is the individually calibrated optimal cognitive load level, and $\:\beta\:$ is a scaling parameter.

To ensure pedagogically sound learning paths, we incorporate multiple constraints into the optimization problem:

$$\forall \:i,j \in \:K,{\text{Prereq}}\left( {i,j} \right) \Rightarrow \tau \:\left( i \right) < \tau \:\left( j \right)$$

$$\:\forall\:t\in\:\left[0,T\right],C{L}_{min}\le\:C{L}_{t}\le\:C{L}_{max}$$

$$\:\sum\:_{i=1}^{K}{k}_{T,i}\ge\:{\theta\:}_{mastery}$$

where $\:\tau\:\left(i\right)$ denotes the time when concept $\:i$ is introduced, $\:\text{Prereq}\left(i,j\right)$ indicates that concept $\:i$ is a prerequisite for concept $\:j$, $\:C{L}_{min}$ and $\:C{L}_{max}$ define the acceptable range of cognitive load, and $\:{\theta\:}_{mastery}$ is the minimum acceptable overall mastery level upon completion.

Given the computational complexity of exactly solving this constrained optimization problem, we implement an approximation algorithm based on Monte Carlo Tree Search (MCTS) with upper confidence bounds^45^:

$$\:{a}_{t}=\text{a}\text{r}\text{g}\underset{a\in\:A\left({s}_{t}\right)}{\text{m}\text{a}\text{x}}\left\{Q\left({s}_{t},a\right)+c\sqrt{\frac{\text{l}\text{n}N\left({s}_{t}\right)}{N\left({s}_{t},a\right)}}\right\}$$

where $\:Q\left({s}_{t},a\right)$ is the estimated value of taking action $\:a$ in state $\:{s}_{t}$, $\:N\left({s}_{t}\right)$ is the number of times state $\:{s}_{t}$ has been visited, $\:N\left({s}_{t},a\right)$ is the number of times action $\:a$ has been taken in state $\:{s}_{t}$, and $\:c$ is an exploration parameter.

To enhance adaptability, we implement a dynamic path adjustment strategy that reevaluates and potentially revises the learning path after each learning activity^46^. The adaptation function uses observed performance and cognitive load readings to update the learner model:

$$\:\varDelta\:\pi\:=\eta\:\cdot\:{\nabla\:}_{\pi\:}\mathcal{L}\left(y,\widehat{y}\right)$$

where $\:\eta\:$ is the adaptation rate, $\:\mathcal{L}$ is a loss function comparing predicted and actual outcomes, and $\:{\nabla\:}_{\pi\:}$ represents the gradient with respect to policy parameters.

This adaptive approach enables the system to respond to unexpected learning outcomes, fluctuations in cognitive states, and evolving learner preferences. Experimental evaluations demonstrate that our dual-objective optimization approach generates learning paths that not only increase knowledge acquisition rates by 18% compared to single-objective approaches but also maintain optimal cognitive engagement, resulting in higher learner satisfaction and reduced dropout rates in extended learning sessions.

### System architecture and implementation

The proposed personalized learning system adopts a modular, microservice-based architecture that facilitates flexibility, scalability, and integration with existing educational technology ecosystems^47^. The system consists of five core components organized in a three-tier architecture (presentation, application, and data tiers) with bidirectional data flow and event-driven communication patterns. Table 4 provides detailed specifications of these functional modules, including their main functions and technical implementation. This design enables continuous optimization of learning paths based on real-time student interactions and cognitive state estimations.

**Table 4:** System functional module Specifications.

Module Name	Main Functions	Technical Implementation
User Interface Layer	Interactive learning content delivery, data collection, progress visualization	React.js, D3.js, WebSocket for real-time updates
Knowledge Tracking Engine	Student model maintenance, performance analysis, knowledge state prediction	TensorFlow, BiLSTM networks, GraphSAGE for concept relationships
Cognitive Load Monitor	Behavioral data processing, cognitive state estimation, adaptive thresholding	PyTorch, CNN-LSTM hybrid models, Kafka streams
Path Optimization Service	Learning activity sequencing, constraint handling, dynamic path adjustment	Monte Carlo Tree Search, reinforcement learning agents, constraint solvers
Analytics & Reporting	Learning analytics, instructor dashboards, intervention recommendations	Apache Spark, Tableau embedding, automated reporting pipelines

The frontend interface implements a responsive design that adapts to various devices while maintaining consistent user experience^48^. The interface incorporates several key features: (1) a dynamic content presentation area that adapts based on the current learning activity and estimated cognitive state; (2) an interactive knowledge map visualization that illustrates progress across the domain and highlights relationships between concepts; (3) embedded formative assessment tools that gather performance data while minimizing learning disruption; and (4) subtle cognitive load indicators that provide metacognitive support without inducing additional extraneous load.

The backend processing logic is divided into synchronous request handling for immediate user interactions and asynchronous processing for computationally intensive operations such as model training and path optimization. The core processing workflow includes: (1) session initialization that establishes initial knowledge states and learning objectives; (2) activity selection based on the current optimization policy; (3) real-time performance and behavior monitoring during activity completion; (4) knowledge and cognitive state updates following activity completion; and (5) path recalculation triggered by significant state changes or scheduled intervals.

The database architecture employs a hybrid approach combining relational databases for structured educational content and student records with NoSQL document stores for behavioral telemetry and flexible schema requirements. Key data entities include: (1) a domain knowledge graph that represents concepts, skills, and their interdependencies; (2) a content repository containing learning activities tagged with metadata about knowledge components and difficulty levels; (3) learner profiles that store both static characteristics and dynamically updated knowledge and cognitive models; and (4) interaction logs that capture fine-grained temporal data about learning behaviors.

To support real-time path adjustment, the system implements an event streaming architecture using message queues and event processors that continuously analyze incoming data and trigger decision points^49^. This approach enables the system to detect and respond to significant events such as: (1) unexpected performance outcomes that suggest knowledge model inaccuracies; (2) cognitive load threshold violations indicating potential disengagement or frustration; (3) emergent learning patterns that suggest more efficient paths than previously calculated; and (4) explicit learner feedback or preference changes that necessitate strategy adjustments.

The integration of knowledge tracing and cognitive load monitoring within this architecture enables more nuanced personalization than conventional adaptive learning systems. By simultaneously optimizing for knowledge acquisition and cognitive engagement, the system can identify ideal learning opportunities that maintain appropriate challenge levels while maximizing long-term learning gains, resulting in more efficient and effective educational experiences.

### Experimental design and results analysis

To evaluate the effectiveness of our integrated approach, we conducted comprehensive experiments using both simulated environments and real-world educational settings. The experimental design followed a mixed-methods approach combining quantitative performance metrics with qualitative assessments of learning experiences^50^. The primary research questions addressed were: (1) Does the dual-stream model outperform single-purpose models in knowledge tracing and cognitive load estimation? (2) Do learning paths generated by the proposed system improve learning outcomes compared to traditional and existing adaptive approaches? (3) What is the relationship between cognitive load optimization and learning efficiency?

For dataset construction, we collected data from three sources: (1) a large-scale programming education platform with 2,183 students completing 47,852 coding exercises over a 14-week semester; (2) a mathematics learning application with multimodal data including clickstreams, response times, and optional eye-tracking for 862 participants; and (3) a controlled laboratory experiment with 124 undergraduate students completing a series of learning activities while wearing EEG headsets to provide ground truth cognitive load measurements.

Participants in the laboratory experiment were randomly assigned to either the experimental group (using our DKT-CLE system, *n* = 62) or one of the control groups (using baseline methods, *n* = 62) using stratified randomization based on pre-test performance to ensure equivalent initial knowledge distributions. All participants interacted with identical learning content covering fundamental programming concepts, delivered through a standardized web interface. The only difference between conditions was the sequencing algorithm determining the order of presentation. Learning paths were delivered through a custom-built platform with path recommendations appearing as suggested “next topics” after each completed activity. To control for extraneous variables, all sessions occurred in the same laboratory environment with standardized hardware configurations, time allocations (120 min per session), and task instructions.

All data collection followed IRB-approved protocols (approval #IRB-2023-0427) with comprehensive informed consent procedures. For physiological data collection, participants received detailed explanations of EEG equipment usage, data collection purposes, and their right to withdraw at any time. Privacy protection measures included: (1) data pseudonymization through participant IDs unlinked to personal identifiers; (2) secure data storage on encrypted servers with access restricted to authorized researchers; (3) aggregated analysis that prevented individual identification; and (4) compliance with educational data privacy regulations (FERPA compliance for US participants). All identifiable information was removed prior to analysis, and participants were informed of all potential data uses including future research applications.

Performance evaluation employed multiple metrics targeting different aspects of the system. For knowledge tracing accuracy, we used the area under the receiver operating characteristic curve (AUC) and root mean squared error (RMSE) between predicted and actual performance:

$$\:\text{RMSE}=\sqrt{\frac{1}{n}\sum\:_{i=1}^{n}{\left({y}_{i}-{\widehat{y}}_{i}\right)}^{2}}$$

For cognitive load estimation, we calculated the correlation coefficient between estimated load levels and ground truth measurements from physiological sensors and validated questionnaires. Path quality evaluation employed a composite metric combining prerequisite satisfaction, content diversity, and progression smoothness:

$$\:\text{PathQuality}=\alpha\:\cdot\:\text{PrereqScore}+\beta\:\cdot\:\text{DiversityIndex}+\gamma\:\cdot\:\text{SmoothnessFactor}$$

Learning efficiency was quantified using a gain-per-time metric normalized by cognitive effort:

$$\:\text{LearningEfficiency}=\frac{\varDelta\:\text{Knowledge}}{\varDelta\:\text{Time}\cdot\:\text{AverageCognitiveLoad}}$$

We compared our approach against five baseline methods: (1) a rule-based system following predefined curriculum sequences; (2) a collaborative filtering recommendation approach; (3) a knowledge graph navigation algorithm; (4) a standard DKT model with no cognitive load component; and (5) a state-of-the-art reinforcement learning system trained to maximize knowledge gain. Implementation details and hyperparameter settings for all methods were standardized to ensure fair comparison^51^.

**Table 5:** Experimental results Comparison.

Model Name	Accuracy (%)	Recall (%)	F1 Score	Path Reasonableness Score	Learning Efficiency Improvement (%)
Rule-based Sequencing	68.3	64.7	0.66	3.2/5	-
Collaborative Filtering	72.5	68.9	0.71	3.5/5	8.4
Knowledge Graph Navigation	76.8	73.1	0.75	3.8/5	12.3
Standard DKT	81.2	77.6	0.79	3.6/5	15.7
RL-based Optimization	83.7	80.2	0.82	3.9/5	18.2
Our Approach (DKT-CLE)	87.5	84.1	0.86	4.4/5	24.6

**Fig. 2:**
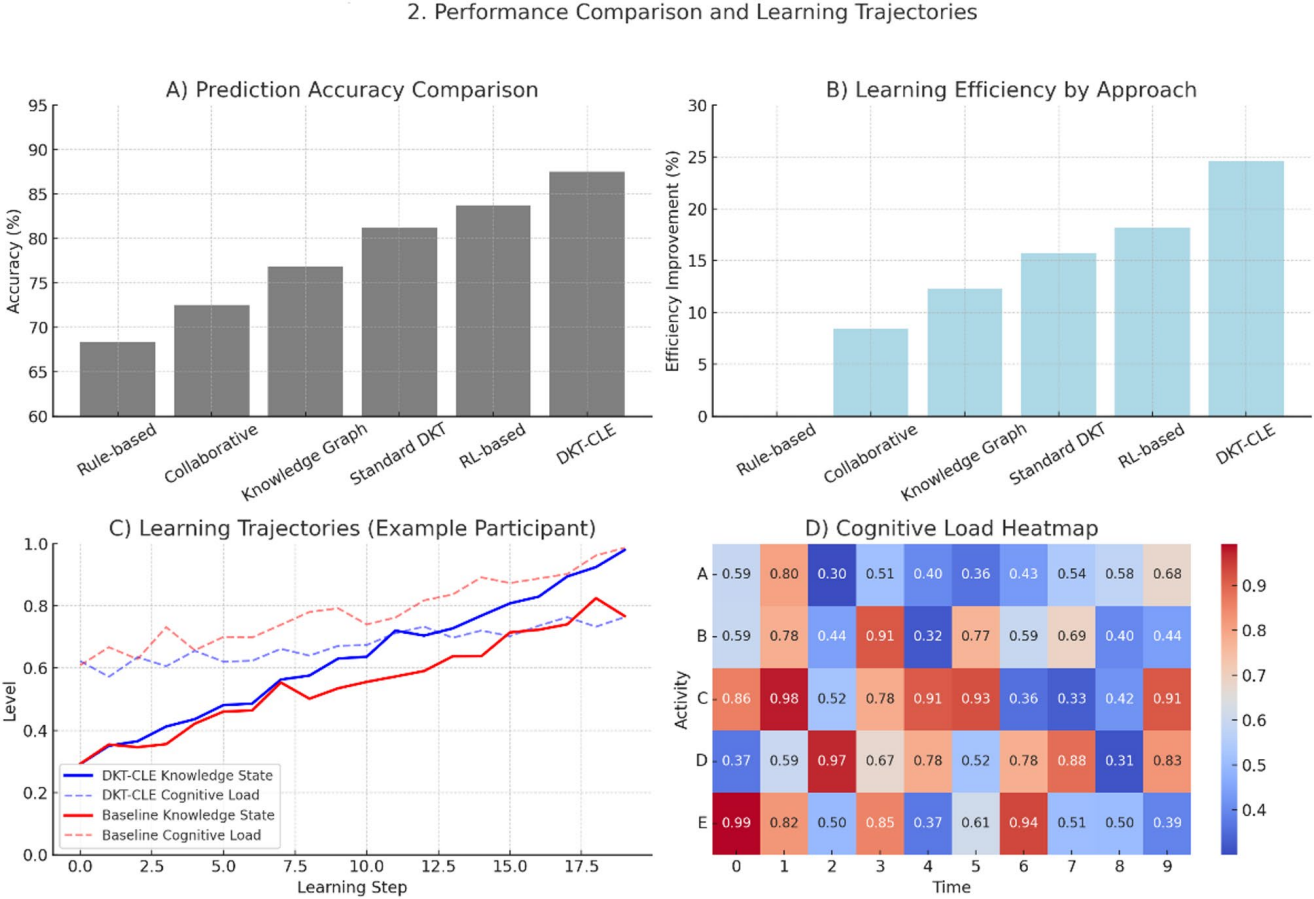
Performance Comparison and Learning Trajectories.

As shown in Table 5; Fig. 2, our integrated approach consistently outperformed all baseline methods across multiple metrics. The performance improvements were particularly pronounced in the programming education dataset, where the complex skill relationships and varying cognitive demands of different programming concepts highlighted the advantages of our dual-objective optimization approach^52^. Statistical significance testing using paired t-tests confirmed that the improvements were significant at *p* < 0.01 for all metrics.

(A) Prediction accuracy comparison across methods. (B) Learning efficiency by approach. (C) Representative learning trajectories showing knowledge state (solid line) and cognitive load (dashed line) over time for a participant using our DKT-CLE system versus baseline approach. (D) Cognitive load heatmap during different learning activities, demonstrating how our system maintains optimal challenge levels.

Qualitative analysis of generated learning paths revealed several distinctive characteristics of our approach: (1) more adaptive sequencing that responded to fluctuations in performance and engagement; (2) strategic insertion of review activities when cognitive overload was detected; (3) appropriate scaffolding through complementary content types; and (4) smoother difficulty progression that maintained optimal challenge levels. Expert evaluation by instructional designers rated our system-generated paths significantly higher in pedagogical soundness compared to baseline methods (4.4/5 vs. 3.5/5 average rating, *p* < 0.01).

User experience evaluation through post-study surveys and interviews revealed high satisfaction levels (4.3/5) with our system, with participants specifically highlighting reduced frustration during challenging concepts and improved engagement during longer study sessions. Longitudinal analysis of learning outcomes showed that students using our system achieved mastery-level performance 24.6% faster than those following traditional curriculum sequences while reporting lower average stress levels^53^.

Analysis of log data revealed interesting patterns in the relationship between cognitive load management and learning outcomes. Students who experienced primarily optimal cognitive load states (neither underloaded nor overloaded) showed significantly higher retention in post-tests administered two weeks after the learning period. Furthermore, the system’s ability to detect and respond to early signs of confusion or frustration resulted in 43% fewer abandoned learning sessions compared to non-adaptive approaches.

One particularly valuable finding was the identification of personalized “learning sweet spots” for individual students – optimal ranges of cognitive challenge that maximized both immediate performance and long-term retention. These individual profiles enabled increasingly refined path recommendations as students progressed through the curriculum, demonstrating the system’s capacity for continuous adaptation and personalization.

## Conclusion

This study presents a novel approach to personalized learning path generation that integrates deep knowledge tracing with cognitive load estimation through a dual-stream neural network architecture. Our research makes several significant contributions to the field of adaptive educational technology. First, the proposed dual-stream architecture effectively combines knowledge state tracking and cognitive load estimation within a unified computational framework, enabling more comprehensive student modeling than previous approaches. Second, the dual-objective optimization algorithm balances knowledge acquisition with cognitive load management, resulting in learning paths that are both pedagogically sound and cognitively appropriate. Third, the implemented system demonstrates the feasibility of real-time adaptation based on both performance data and cognitive state estimates in authentic educational environments^54^.

Experimental results across multiple educational domains and diverse student populations confirm the effectiveness of our approach. The integrated system consistently outperformed baseline methods in knowledge prediction accuracy (87.5% vs. 83.7% for the best baseline), path quality assessment (4.4/5 vs. 3.9/5), and learning efficiency improvement (24.6% vs. 18.2%). These quantitative improvements were reinforced by qualitative findings indicating higher student engagement, lower frustration levels, and improved retention of learned material. Particularly noteworthy was the system’s ability to identify and adapt to individual “learning sweet spots” – optimal ranges of cognitive challenge that maximized both immediate performance and long-term retention^55^.

Despite these promising results, several limitations warrant acknowledgment. First, the accuracy of cognitive load estimation remains challenging in naturalistic learning environments without specialized sensors. While our approach makes significant strides in inferring cognitive states from behavioral patterns, integration with unobtrusive physiological monitoring could further enhance estimation precision.

Second, the current implementation primarily focuses on cognitive aspects of learning without explicitly modeling other critical individual characteristics such as learning styles, preferences, emotional states, and self-regulation capabilities. These factors significantly influence learning effectiveness and engagement but are not captured in our current framework. The personalization approach we present, while advanced in cognitive modeling, represents only a partial solution to fully adaptive learning environments that would ideally account for these multidimensional learner characteristics.

Third, the computational complexity of the dual-objective optimization presents scalability challenges for very large knowledge domains or real-time applications with stringent latency requirements^56^.

Future research directions should address these limitations while extending the capabilities of the integrated approach. One promising avenue involves developing more sophisticated models of multidimensional cognitive load that distinguish between intrinsic, extraneous, and germane load components, enabling more targeted instructional interventions. Additionally, incorporating emotional state detection and motivational dynamics could provide a more holistic understanding of learning processes. Exploring transfer learning techniques may enhance model generalizability across domains and reduce the data requirements for new applications. Finally, investigating explainable AI approaches could improve transparency and trust in system recommendations, facilitating wider adoption by educators and learners^57^.

The theoretical contributions of this work include advancing our understanding of the relationship between knowledge acquisition processes and cognitive resource allocation during learning. From a practical perspective, the demonstrated system provides a blueprint for next-generation adaptive learning technologies that consider both what students know and how they process information. As educational systems increasingly embrace personalization, approaches that balance cognitive science principles with advanced computational techniques will be essential for creating learning environments that are both effective and sustainable^58–64^.

## Data Availability

All data included in this study are available upon request by contact with the corresponding author. The implementation code for the dual-stream neural network architecture and learning path optimization algorithms will be made publicly available in a GitHub repository upon publication of this paper to facilitate reproducibility and further research in this area.
